# A qualitative study of the current situation of elderly care in Iran: what can we do for the future?

**DOI:** 10.3402/gha.v9.32156

**Published:** 2016-11-21

**Authors:** Salime Goharinezhad, Mohammadreza Maleki, Hamid Reza Baradaran, Hamid Ravaghi

**Affiliations:** 1 School of Health Management and Information Sciences, Iran University of Medical Sciences, Tehran, Iran; 2 Health Services Management Research Center, Institute for Futures Studies in Health, Kerman University of Medical Sciences, Kerman, Iran; 3 Health Management and Economics Research Center, Iran University of Medical Sciences, Tehran, Iran; 4 Endocrine Research Center, Institute of Endocrinology and Metabolism Iran University of Medical Sciences Tehran, Iran

**Keywords:** elderly care, current situation, challenge, qualitative study, Iran

## Abstract

**Background:**

With the successful improvement of global health systems and social security in societies, the world is now advancing toward aging. All countries have to face the phenomenon of population aging sooner or later depending on their degree of development; however, elderly care is predicted to soon become a major concern for developing countries such as Iran.

**Objectives:**

This study was conducted to identify the challenges of elderly care in Iran and to help policymakers develop roadmaps for the future through providing a clearer image of the current state of affairs in this area of healthcare.

**Design:**

This study has adopted a framework approach to qualitative data analysis. For this purpose, 37 semi-structured interviews were conducted in 2015 with a number of key informants in elderly care who were familiar with the process at macro-, meso-, and micro-levels. Maximum variation purposive sampling was performed to select the study samples. A conceptual framework was designed using a review of the literature, and key issues were then identified for data analysis.

**Results:**

The elderly care process yielded five major challenges, including policymaking, access, technical infrastructure, integrity and coordination, and health-based care services.

**Discussion:**

According to the stakeholders of elderly care in Iran, the current care system is not well-suited for meeting the needs of the elderly, as the elderly tend to receive the services they need sporadically and in a non-coherent manner. Given the rapid growth of the elderly population in the coming decades, it is the authorities’ job to concentrate on the challenges faced by the health system and to use foresight methods for the comprehensive and systematical management of the issue.

## Introduction

With the beginning of the third millennium, the phenomenon of aging has become more and more manifested as a major global event (1, 2). Population aging is a byproduct of the development of societies and indicates that the former human efforts for the treatment and control of epidemic diseases, on the one hand, and the low birth rates, on the other, have put the world’s population on the verge of aging (3). Paul Wallace, the author of Agequake, writes (4), ‘Historically, we have been remarkably young. Our average age has been around 20 or less. But in the current generation’s lifetime, the average age of the world will nearly double from 22 in 1975 to 38 in 2050, According to the UN projections. Under another projection, it could reach over 40 as early as 2040’, which is the first time in history that the elderly population overtakes the young population (5). The increasing rate of population aging is a common problem in both developed and developing countries; however, the rate is massively different between the two (6). In France, for example, the elderly population will reach 14% from the current 7% in a matter of 115 years, while the elderly population has in fact doubled over the past two to three decades in developing countries such as Thailand, Brazil, and China (7).

According to the official predictions of international organizations, as a developing country, Iran is also approaching population aging (8, 9). The United Nations report of 2012 warns that, along with Mongolia and Cuba, Iran will experience the most rapid demographic changes (10). Preemptive measures are therefore required to be taken immediately to overcome this phenomenon. Just as in the rest of the world, the phenomenon of population aging in Iran is also a result of increased life expectancy, improved health, and a general reduction in fertility (8). In the 1960s, Iran’s population control policy aimed Iranians with the slogan of ‘Fewer children, better life’ and resulted in reduced birth rates (11). The family planning program was later discarded due to the prolonged Iran–Iraq war and the need for young efficient workforce and thus led to a population explosion in the 1980s (12). This population explosion highlighted the needs of children and adolescents (e.g. nutrition, immunization, health, and schooling) throughout the 1980s and 1990s in the government’s macro-plans. From 2000, meeting the needs of the youth (e.g. employment, housing, and marriage) became the main concern of government officials and policymakers (13). Elderly affairs were therefore marginalized and not incorporated into the national policies in spite of Iran’s rich culture of respecting the elderly and the great emphasis placed in religious teachings on honoring senior citizens (14). Evidence suggests that, in the Iranian planning and policymaking system, the history of political debate on the issue of aging does not exceed a decade, and there is still a lack of factual evidence based on analyses of the country’s situation for enabling correct decision-making and planning. Despite the fact that older adults need care services, little direct attention has been paid to providing care for them. Since the phenomenon of aging will have a significant impact on shaping care patterns and demands related to the healthcare system in the future, ethics mandates taking urgent measures to deal effectively with this phenomenon in future. Therefore, this study aimed to determine the current status of elderly care in Iran and identify its challenges to help policymakers develop and organize a comprehensive elderly care system based on reality.

## Methods

A qualitative method was used to obtain deep and rich information from participants regarding the study objective to identify the current status of elderly care. Since this study was based on the World Health Organization policy framework on active aging (15), framework analysis was used to analyze the data. This method has recently become popular in the field of health and research related to policymaking and health services (16).

### Sampling

The study participants were selected from the stakeholders who had enough experience and expertise on the phenomenon in question. The main inclusion criteria consisted of having experience in and expertise on aging and health policymaking and giving consent to participation in the study. A total of 37 candidates were included in the study using maximum variation purposive sampling. Sampling continued until data saturation and until no further information and classes were being extracted.

### Data collection tools

The data collection tools used included semi-structured interactive interviews in individual and sometimes group formats. The interview guide questions were designed based on a review of literature and using the comments made by informed advisers. Pilot interviews were then conducted with two experts, and their deficiencies were then resolved in the research team. The interviews were conducted in the workplace of the participants after setting an appointment with them and once the face and content validities of the interviews were confirmed. Before beginning the interviews, the participants were ensured about the complete voluntary nature of participation in the study and their right to withdraw from it at any stage. The duration of the interviews varied from 15 to 75 min. After conducting and recording each of the interviews, their audio files were immediately transcribed verbatim, and an initial analysis of their content was then performed to provide a guide for continuing the data collection and analysis process. The researcher recorded any uncertainties or questions that arose through the review of the data and then followed up on them in telephone interviews or in the interviews held with the next participants.

### Data analysis method

The framework analysis method, used to analyze the data, consists of five steps, namely familiarization with the interview, developing a working analytical framework, indexing, charting, and interpreting the data (16). In the familiarization step, preliminaries were provided for more familiarization and immersion in data by listening to recorded interviews and reading them several times, and the key themes were listed. In the second step, a thematic framework of the key topics was prepared based on the WHO framework, which was used in the indexing stage for structuring all the data. In the charting step, a table was drawn for each theme, and the data were transferred to it. In the interpretation step, the relationship among codes, subthemes, and themes was described.

### Trustworthiness

Two professors and qualitative research experts (MM and HR) verified the credibility of the data through the accurate and stepwise control of the research process. Interview texts were also sent to the participants with the initial codes extracted so as to comment on their authenticity and to enhance the transferability of the extracted data. The confirmability of the data was ensured by interviewing a very different selection of participants in terms of age, gender, expertise, experience, level of education, and level of service delivery and also through the frequent review of the data. The dependability of the data was confirmed by the audit trail. For this purpose, meaning units, codes, sub-themes, and themes were reviewed by an independent researcher.

## Findings

**Fig. 1:**
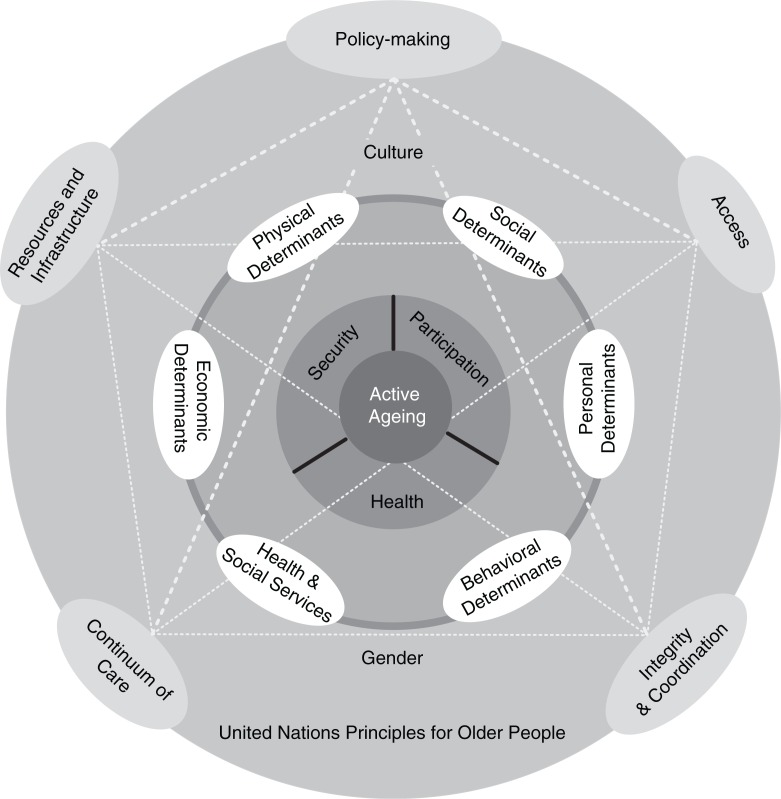
Conceptual framework to achieve active aging goals in developing countries.

The study participants consisted of 37 stakeholders at different executive levels. Table 1 presents their composition in terms of level and executive position. This stage of data collection lasted from May to December 2015. The careful review of the interviews and the extraction of conceptual units as codes yielded a total of 614 initial codes. Through the constant comparison and analysis of the data, the codes were placed into 5 main categories and 16 subcategories comprising the challenges of elderly care in Iran. Table 2 presents the distribution and formation of the categories. Figure 1 showed that five key themes in relation to active ageing policy framework. in fact any measures to achieve active ageing will need these prerequisites.

**Table 1:** The composition of the study participants

Stakeholders’ executive level	Executive position	Number
Superior level (management and	The Ministry of Health and Medical Education	6
policymaking at the national level)	The Ministry of Welfare and Social Security; the Welfare Organization of Iran	5
Macro	The Municipality Organization and Imam Khomeini Relief Foundation	3
	Academy of Medical Sciences	2
Colleague level (faculty members and	Geriatric Medicine and Geriatrics Faculty Members	5
researchers)	Researchers	2
Meso	Other faculty members	3
Subordinate level (executive-level staff)	Healthcare centers	6
Micro	Nursing homes	3
	Senior associations	2
Total number of participants		37

**Table 2:** Challenges of elderly care in Iran

Theme	Sub-theme
Challenges in policymaking	Stewardship and governance
	Content of the policies
	Actors and stakeholders
Challenges in access	Geographical access
	Economic access
	Cultural access
Challenges in technical resources	Human resources
and infrastructure	Physical resources
	Information resources
Challenges in integrity and	Intrasectoral coordination
coordination	Intersectoral coordination
Challenges in continuum of care	Primary healthcare
	Informal and family-based care
	Community-based care
	Medical care and rehabilitation
	Long-term palliative care

### Theme one: challenges in policymaking

The stewardship and governance of elderly care were one of the sub-themes emerging in this category. According to the participants, there is no fixed trusteeship for elderly care services at the present moment and there are conflicts between the Ministry of Health and the Ministry of Welfare over providing elderly care services, thus leading to sporadic disunited actions:

It is not yet properly known which institution is the trustee for providing services to the elderly. The legislator has entrusted the Welfare Organization with the job of looking after seniors with disabilities and the Ministry of Health with the job of looking after the health of the elderly, having thus led to an ambiguity of roles. (P9)

You can practically see for yourself that the Ministry of Health has presented one document or factsheet and the rehabilitation organization another totally irrelevant sheet. (P3)

In addition to the lack of a fixed trusteeship and governance, weaknesses in the content of the developed policies was another theme emerging in this category. The participants described the policies developed on aging to be idealistic, treatment-based, ideology-based, lacking legal sanction, and oblivious to the infrastructures and prerequisites, stipulating that there are no clear policies developed for comprehensive elderly care in Iran and that the majority of the existing programs and policies are temporary and do not cover all the aspects of elderly care.

Our policies are so idealistic that have already failed, as the program prerequisites have not been considered in their drafting and since they lack an enforcement guarantee. (P18)

Policies are mostly developed based on beliefs and ideologies, emerging when one minister holds office and forgotten when they relinquish from that position. (P16)

Health policies are more concerned with the treatment than with the health dimension of healthcare, and health ministers and policymakers, most of whom are physicians, have an often therapeutic vision. (P7)

The role and power of stakeholders and actors in setting targets and policy orientations was another theme emerging in this category. Many experts identified the collaboration between stakeholders and actors as a significant factor contributing to the successful establishment and implementation of elderly care policies. ‘The successful implementation of elderly care depends on an ambiance of cooperation and interaction between the Ministries of Health and Welfare’ (P2). They also believed that the government alone cannot meet all the needs of the elderly, as this group of the population has diverse needs that necessitate the cooperation of the private sector, charity organizations, and municipalities as actors.

We should not rely only on the government for dealing with problems such as aging. Unfortunately, the government has always been the mainstay of our plans and we have always waited to see what plans and measures the government adopts for handling these issues, and this is troublesome. If we link the life of our elderly to the ups and downs of governments, we will accomplish nothing, as is the case now that we have failed to achieve the desirable outcomes. It is crucial to take advantage of what municipalities, charity organizations, and NGOs have to offer. (P12)

### Theme two: challenges in access

The verbal statements led to the abstraction of three subcategories, including geographical, economic, and cultural access. The unfavorable economic status of the elderly was one of the main problems stated in this category. According to the participants, the small old age pensions and the lack of a source of income for many of the elderly and basic insurance policies’ failure to cover the costs of many crucial elderly care services have made the utilization of age-specific services a difficult task for the elderly: ‘Many services that the elderly needs are not covered by basic insurance policies; they thus need to take supplementary medical insurance’ (P12). ‘This group of the society has such low economic status that they are unable to even make ends meet. How can we expect them to take expensive screening tests?’ (P20).

In the geographical dimension, the participants noted the lack of age-friendly environments and transport facilities and the accumulation of facilities in metropolises. ‘In our country, some of the elderly do not have access to services because they are homebound! How can they access these services then? This group should have been given special facilities, but this is not the case now’ (P3). ‘The vast majority of those who lived in villages and suburban areas in deprived areas of Iran are deprived of specialized health services in spite of paying premiums equal to those paid by the citizens of metropolises’ (P19). Personal beliefs, illiteracy, especially health illiteracy, and lack of awareness about the available services are among the cultural obstacles against the elderlies’ access to services. ‘Some women cannot show up in society due to religious beliefs. For example, there have been cases in which sick women have not presented to receive services because the center physician was male’ (P19). ‘Approximately 70% of the Iranian elderly are illiterate, which undoubtedly affects their awareness about the access they can have to care services’ (P1).

### Theme three: challenges in technical resources

The participants believed that a trained workforce is the main infrastructure required for the implementation of elderly care programs at all levels and that Iran is faced with serious deficiencies in this regard. They argued that, despite the announcements made about integrated elderly care programs in urban and rural health centers, the infrastructures required for the execution of the program are lacking, including the required human, physical, and information resources, which is why the program has not been welcomed by the target group.

In our health centers, the expert workforce has to simultaneously handle several jobs. For example, they should perform vaccinations, do a check-up of pregnant women, take care of family planning and dedicate at least 45 minutes to the screening of each elderly based on the booklet instructions. They should also manually report the screening, which is itself a time-consuming task. More importantly, there are no dedicated spaces for examining the elderly, and they should be examined in the same place where children are shouting and making noises, which is by no means desirable for the elderly. (P3)

The lack of resources is also discernible at the second and third levels of care. According to the participants, geriatricians are rare, and there are no geriatric clinics in the country; however, even though

The number of geriatricians is less than a handful, the shortage is not limited to specialists, but involves other workers as well, such as nurses, health experts, pharmacists and dentists specializing in elderly health. (P17)

In spite of the significant advances in different medical sectors in Iran, the establishment of specialized centers for the elderly has grown but very little. We currently have three specialist children’s hospitals in Tehran, and it is only reasonable to have a proportionate number of geriatric hospitals, as it is the same children who are now growing old. (P13)

Any measures or decisions taken for the elderly should be based on reliable information about their personal and social characteristics. The participants identified the lack of a comprehensive information system for the elderly as one of the challenges in planning and policymaking, stipulating that Iran is not ranked by the Global AgeWatch for lack of information.

We do not even have one database of our elderly. Our data on aging is so weak that we are not ranked at all by the HelpAge International or the Global AgeWatch and no institutions have yet collected this data. As long as realistic data are not available, taking any actions is like pouring water into a sieve. (P8)

### Theme four: challenges in integrity and coordination

Many experts emphasized the importance of creating and maintaining integrity and coordination between different levels of the health system and also between the health sector and the other sectors of the society. Reinforcing the referral system and the relationship between different levels of healthcare and launching an electronic health record system are major components of intrasectoral coordination.

There are no well-defined referral systems in the integrated elderly care program proposed, and our elderly do not have easy access to specialized services, as they are simply given a referral paper to take with themselves whenever they visit a doctor. Developing an information system that coordinates and integrates services between different health centers and hospitals can fix the issue and help define coherent relationships between the prevention, treatment and rehabilitation levels of healthcare. (P23)

The challenges in intersectoral coordination include the weaknesses of coordination and interaction between the institutions that provide social, legal, and health services. Given that the elderly’s care needs comprise different dimensions, centers providing health services should be well coordinated with social support centers.

In our screenings, we came across severely undernourished elderly patients. Our investigations revealed their extreme poverty and even an inability to buy fruits, but we could not help them anyways, as we are not in a position to introduce them to the Welfare Organization or to Imam Khomeini Relief Foundation. (P30)

### Theme five: challenges in continuum of health services

According to the WHO active aging policy framework, as the population ages, all countries need a coordinated and comprehensive continuum of care with more emphasis on health promotion and preventive care (15). Despite this issue, in Iran, the primary healthcare is not the gateway for access to other needed services. According to the experts, one of the main problems in primary healthcare was that it received less attention compared to hospital services.

I think that the health system should change in terms of both policymaking and implementation. The Ministry of Health is currently focused on treatment programs rather than on health. Our health ministers have always been more treatment-oriented than health-oriented and a major portion of the Ministry budget is spent on treatment matters pertaining to the public. I think that, as long as this viewpoint prevails, health programs will fail. (P7)

The inadequate financial support and the lack of training courses and empowerment programs for unofficial home caregivers are the major problems in this area. The participants believed that the subsidy paid by the government for elderly care is too little to meet the elderly’s diverse needs.

Unfortunately, in Iran, the government’s support for families with handicapped elderly is not enough to meet their every needs. Plus, these families need special training courses that teach them how to take proper care of their elderly. (P19)

Community-based services for the elderly include nursing homes or adult daycare services, clubs, cultural centers, meals on wheels, and transportation services for the vulnerable, frail, and disabled elderly. These centers provide medical and social services to the elderly and play a key role in the maintenance of their independence, better plans for leisure time, and participation in the community.

The experts argued that the number and nationwide distribution of these centers is inadequate and unfair. In addition, the services they provide do not abide by international standards and are designed mainly for the elderly with disabilities.

Daycare and respite care centers help families with their elderly care duties. However, these centers are very rare and even then are mostly found in metropolises. Most importantly, the services they provide lack the required variety and are often limited to routine services fit for the elderly with diseases. It is a must for these centers to incorporate educational, cultural and art programs into their schedule in addition to providing medical services. (P25)

Investigating the hospital and medical care provided for the elderly showed that, not only do hospitals lack systems for assigning priority levels to their elderly patients but the medical team also commits a stereotypical error known as ageism in their care processes, which causes the elderly’s diseases to be attributed to their aging and to thus be given lower priorities than the diseases of the young. The participants also noted that hospital ambiences were not age-friendly, that there were no geriatric clinics or units for the comprehensive examination of the elderly and also that the lack of electronic health records made it difficult to obtain the elderly’s medical history and the treatment process was therefore often slowed down and impeded.

Not only don’t we have a system for assigning priority levels, but the elderly patients are often not taken seriously enough by the medical team. The society has an unfair view of the elderly, as they tend to believe that the elderly have passed the productive stage of their life and are no longer needed by the society. The last priority is given to them for the receiving of services as a result of this perception. (P15 and P4)

In Iran, hospitals are not customized for the elderly, whether in terms of their physical space or the care services they provide to them. An elderly patient may present to the hospital with several diseases and visit a cardiologist, a neurologist, an internist, a pulmonologist and a psychologist. These specialists prescribe medications for the treatment of the one disease they have diagnosed, but the simultaneous consumption of sometimes more than ten medications may pose side effects for the elderly patient. Had there been a geriatrician, the patient would have received a comprehensive examination instead. (P10)

The lack of electronic health records make the diagnosis and treatment process more difficult for the illiterate elderly or the elderly with amnesia, as the physician has to ask the patient so many different questions to obtain, if possible, the initial patient information, which is both time- and energy-consuming. (P14)

Discharging the elderly from the hospital without proper follow-up plans or referrals to post-treatment rehabilitation services was another shortcoming of the elderly health services provided in Iran.

There are no links here between preventive medicine, therapeutic medicine and rehabilitation medicine. For example, if an elderly patient suffers a stroke and is paralyzed, they are discharged after they feel better without ever being sent to the rehabilitation unit or being followed up on. The outcome of this neglect is bedsore, getting crippled, joint stiffness and depression, which gradually destroy the patient. (P26)

Concern with the quality of the services provided in nursing homes was another problem emerging in the category of health-based care. According to the participants, nursing home services should be constantly monitored in terms of the ratio of trained personnel to elderly, physical space and environment standards, financial resources and social collaboration programs, and the quality of services should also be consistently enhanced.

The services provided in nursing homes should be inspected in terms of human resources, service quality, safety and social collaboration programs and certain standards should be developed and consequently enforced. (P33)

The lack of palliative and end-of-life care for the terminally ill elderly was also another shortcoming of the elderly health services provided in Iran. The participants declared that palliative and supportive care were not yet fully incorporated into the national health policies and that raising awareness about the issue was necessary.

Ideally, care centers such as hospitals, residences and hospices should use palliative care for the control of pain in terminally ill patients. Nevertheless, palliative medicine literature has recently entered the health system and requires more time to be further recognized. (P29)

## Discussion

This study was conducted to identify the main challenges in elderly care in Iran by asking key informants of the field. Research suggests that concerns with the issue of elderly care and the adoption of proper policies on the subject are global; however, the problem is more urgent in developing countries, as these countries have less time to adapt to the consequences of the phenomenon of aging (17, 18). The results obtained in this study showed that the national elderly care policymaking in Iran is substandard and that the two main actors of elderly care, that is, the Ministry of Health and the Ministry of Welfare, are in conflict with each other and do not interact adequately well. In other words, depending on their power and authority, each of the ministries identifies itself as the main trustee in this area. The National Seniors Council’s inactive status after several years from its regulatory approval further supports this claim. Ahmadi (19) described uniform policymaking for the elderly care as a crucial factor in the development of care programs, the direction of resources, and the accomplishment of senior health targets.

Another challenge identified in elderly care services was the elderly’s access to the services. A study conducted on elderly care in India shows that a group of trained health personnel have been employed in this country to pay home visits to the elderly so as to facilitate their access to preventive services in the primary healthcare system (20). The findings of this study, however, revealed that homebound elderly with low activities of daily living levels are deprived of health services, and no facilities have been developed to facilitate their access to health services. The unfavorable economic status of the Iranian elderly was another obstacle against the elderly’s access to health services. In support of this finding, the Iranian Parliament’s report on the economic status of the elderly found that around 25% of the Iranian elderly have no insurance coverage and also that basic insurance plans do not cover the majority of the care services required by the elderly, which further limit the elderly’s access to essential health services (21). A review of EU countries’ experiences reveals that their senior citizens have full coverage for their health services (22). In Iran, the increasing costs of treatment and rehabilitation necessitate the design of full-coverage senior health insurance.

According to the results obtained, Iran is faced with shortcomings in the physical, human, and informational infrastructures and resources required for providing proper elderly care at all levels of the system. The experiences of the health center experts indicate the failure of integrated elderly care programs at the health center level, as these centers have been designed and structured in the distant past for providing maternal and child healthcare, and in their original capacity, they have provided quite effective services too; however, their facilities, resources, and infrastructures are short of meeting the diverse needs of the elderly. Studies reveal similar conditions in many low- and middle-income countries, in which the current structures do not meet the requirements of providing elderly services (23–26). The results of this study also show that a skilled geriatric workforce, which is the main component of elderly care, is scarce in Iran and the few that exist cannot meet the demands of the country’s elderly population. Evidence suggests that developed countries have embarked on the training of home care providers so as to compensate for a portion of the human resources deficiency in elderly care (27). Training an intermediate and skilled workforce, therefore, seems an effective solution in developing countries as well.

An important component of elderly healthcare services emphasized and redefined by the WHO is the principle of ‘integrity and coordination of health and social services’, which holds that, in and by itself, health cannot promote active aging and that all the elements of the society need to contribute their share (15). This study shows that the elderly receive care services in an uncoordinated, sporadic, and even confusing manner and that there are no definite relationships between the health services and the social services that they receive. The WHO Regional Office for the Eastern Mediterranean has suggested elderly care to be provided as a general PHC-based model incorporating socioeconomic services, which is to be implemented in the Eastern Mediterranean Regional Office of the World Health Organization (EMRO) countries by 2021 (28). The comprehensive continuum of care comprised another theme of the study findings. The analysis of the findings obtained showed that the Ministry of Health is focused on treatment rather than on prevention and that a major portion of Iran’s health budget is spent on hospitals and therapeutic services. A 2008 WHO report entitled ‘Primary Health Care: Now More than Ever’ indicates that a large part of the financial resources of the health system is spent on costly treatment services, while prevention and health promotion can reduce the global burden of diseases by up to 70% (29). In order to be able to respond to the altered pattern of diseases and control non-communicable diseases and the increased burden of these diseases as well as other psychosocial problems, the health system needs to adopt an approach of ‘aging and life course’. Instead of a mere attention to medical care, this approach emphasizes the importance of social interactions, self-care, and the necessity of social support for the independent living of the elderly by their families’ side. Community-based care centers such as adult day service and respite care centers are support structures that play a key role in guaranteeing healthy aging. The results of this study also show that such structures are not thriving in Iran and that they are not built to provide diverse medical, educational, and support services. According to a UNFPA report on Iran, one of the reasons for which these centers have not been properly accepted and developed in Iran is that their costs are not covered by either basic or supplemental insurance. The report also reveals that elderly care services in Iran are devoted to taking care of the elderly with disabilities (14).

In examining the treatment and rehabilitation services provided to the elderly, this study found that the elderly admitted to hospitals and health centers receive services just as any other age group, with no dedicated elderly treatment units available in hospitals in Iran; the number of gerontologists and experts in relevant fields of healthcare is alarmingly low; and no comprehensive geriatric assessments are performed on the elderly. Evidence showed that for the elderly who were admitted to the unit for acute care of the elderly, the duration of stay was shorter and the functional capabilities were better, compared with the elderly admitted to general hospital departments (30, 31). Given the increase of elder people in Iran, developing and equipping hospitals to geriatric care units and elderly wards will be a significant need.

The results obtained show that the services provided in nursing homes in Iran do not meet the required standards and that living in these centers is quite inert and routine and the care plans adopted in them are also not designed individually to match the conditions of each elderly and are a mere cutting of corners. Studies show that, given the growth in urbanization, immigration and female employment rates, the formation of nuclear families, and the attenuation of family support networks, an increase in the demand for nursing homes is inevitable in the future. Enhancing the quality of nursing homes and matching them with the future needs and expectations of the elderly are therefore crucial at this stage (32, 33). Nevertheless, these interpretations should be carried out with greater caution, as the acceptance or rejection of this issue depends entirely on the cultural background and the particular conditions of each society.

Although this study tried to obtain a reasonable approximation of the current status of elderly care system in Iran based on the views of a group of policymakers and executives, this study alone cannot express all facts related to elderly care system and provide a definitive answer to all questions about developing an efficient elderly care system. There are still many unknown issues about how an elderly care system works or its slow response to the growing needs. Therefore, conducting further research and surveying the viewpoints of older people about elderly care is also suggested.

## Conclusion

The present elderly care system in Iran was found to provide the elderly with their required services in a sporadic, uncoordinated, and non-coherent fashion. If policymakers decide to organize an efficient and effective system for the large population of future older adults, they will need a clear understanding of the key challenges of the current status of elderly care system. Therefore, this study offers reliable evidence to policymakers by providing a qualitative methodology utilizing the views and perspectives expressed by implementers for challenges. The results of this study can help policymakers in developing countries to choose reasonable options for effective interventions so that older adults can benefit from a healthy and dignified life in future.

## Paper context

There are limited studies about challenges of the health system preparedness with respect to aging population, especially in developing countries like Iran. Therefore, this research is a good starting point for any performance promotion effort targeting elderly care on a national level in the developing countries. The findings of this study are valuable because they provide baseline information toward WHO active aging policy framework implementation. In fact, without attention to five key elements which arise from this qualitative research, the government would have difficulty in meeting the high-quality healthcare and aged-care services.

## References

[1] Davidson PM, DiGiacomo M, McGrath SJ (2011). The feminization of aging: how will this impact on health outcomes and services?. Health Care Women Int.

[2] Christensen K, Doblhammer G, Rau R, Vaupel JW (2009). Ageing populations: the challenges ahead. Lancet.

[3] Beard JR, Officer A, de Carvalho IA, Sadana R, Pot AM, Michel J-P (2016). The World report on ageing and health: a policy framework for healthy ageing. Lancet.

[4] Wallace P (1999). Agequake.

[5] United Nations (2013). Department of Economic and Social Affairs Population Division. World Population Ageing.

[6] Lloyd-Sherlock P (2000). Population ageing in developed and developing regions: implications for health policy. Soc Sci Med.

[7] WHO (2003). Ageing and health: a health promotion approach for developing countries.

[8] Kiani S, Bayanzadeh M (2010). The Iranian population is graying: are we ready?. Arch Iran Med.

[9] Noroozian M (2012). The elderly population in Iran: an ever growing concern in the health system. Iran J Psychiatry Behav Sci.

[10] Ahmadi A, Seyedin H, Fadaye-Vatan R (2015). Towards age-friendly hospitals in developing countries: a case study in Iran. Health Promot Perspect.

[11] Tober DM, Taghdisi MH, Jalali M (2006). ‘Fewer children, better life’ or ‘as many as god wants’?. Med Anthropol Q.

[12] Karamouzian M, Sharifi H, Haghdoost AA (2014). Iran’s shift in family planning policies: concerns and challenges. Int J Health Policy Manag.

[13] Roudi-Fahimi F (2002). Iran’s family planning program: responding to a nation’s needs.

[14] Khosravi A, Alizadeh M, Torkashvand M, Aghaei N (2014). Population ageing in IR Iran.

[15] WHO (2002). Active ageing: a policy framework.

[16] Ritchie J, Spencer L (2002). Qualitative data analysis for applied policy research. Qual Res Comp.

[17] Goharinezhad S (2016). Foresight of elderly care in Iran: a scenario approach. PhD dissertation.

[18] Rechel B, Doyle Y, Grundy E, McKee M (2009). How can health systems respond to population ageing?.

[19] Ahmadi S (2015). Elderly health policy analysis in Iran.

[20] Verma R, Khanna P (2013). National Program of Health-Care for the elderly in India: a hope for healthy ageing. Int J Prev Med.

[21] Health Commission of Parliament (2009). Strategies to support the elder people in Iran.

[22] Rechel B, Grundy E, Robine J-M, Cylus J, Mackenbach JP, Knai C (2013). Ageing in the European union. Lancet.

[23] Phillips DR (2002). Ageing in the Asia-Pacific region: issues, policies and future trends.

[24] Gómez F, Curcio CL, Duque G (2009). Health care for older persons in Colombia: a country profile. J Am Geriatr Soc.

[25] Akanji BO, Ogunniyi A, Baiyewu O (2002). Healthcare for older persons, a country profile: Nigeria. J Am Geriatr Soc.

[26] Montero-Odasso M, Przygoda P, Redondo N, Adamson J, Kaplan R (2004). Health care for older persons in Argentina: a country profile. J Am Geriatr Soc.

[27] Dooghe G (1992). Informal caregivers of elderly people: an European review. Ageing Soc.

[28] WHO (2006). A strategy for active, healthy ageing and old age care in the Eastern Mediterranean region 2006–2015.

[29] WHO (2008). The World Health Report 2008: Primary health care – now more than ever.

[30] Barnes DE, Palmer RM, Kresevic DM, Fortinsky RH, Kowal J, Chren M-M (2012). Acute care for elders units produced shorter hospital stays at lower cost while maintaining patients’ functional status. Health Aff.

[31] Palmer RM, Landefeld CS, Kresevic D, Kowal J (1994). A medical unit for the acute care of the elderly. J Am Geriatr Soc.

[32] Duffy JA, Duffy M, Kilbourne WE (2001). A comparative study of resident, family, and administrator expectations for service quality in nursing homes. Health Care Manag Rev.

[33] Vincent JA, Phillipson C, Downs M (2006). The futures of old age.

